# A miniaturized and low-cost atmospheric pressure helium plasma jet device with high antimicrobial efficiency

**DOI:** 10.1016/j.eti.2026.104852

**Published:** 2026-03-09

**Authors:** Jingqin Mao, Thomas P. Thompson, Laura A. McClenaghan, Ross M. Duncan, Brendan F. Gilmore, Hamza Shakeel

**Affiliations:** a School of Electronics, Electrical Engineering and Computer Science, Queen’s University Belfast, Belfast BT7 1NN, United Kingdom; b School of Pharmacy, Queen’s University Belfast, Belfast BT7 1NN, United Kingdom

**Keywords:** Non-thermal plasma, Dielectric barrier discharge, Atmospheric pressure plasma jet, Antimicrobial, Plasma medicine

## Abstract

This paper reports an atmospheric pressure non-thermal plasma jet device (mini plasma jet) for antibacterial application. The device includes a glass tube, two copper ring electrodes, a custom-made power supply, a helium supply source, and flow control system. The power supply in our device is smaller and lighter compared to other commercially available power supplies. The plasma jet tube is made using a standard glass tube with a narrow outlet. We also simulated the electric field distribution inside and outside the glass tube using COMSOL Multiphysics and optimized the ring electrode distance based on the simulation results. The final system including the power supply and glass tube costs less than U.S. $45. Additionally, our mini plasma jet demonstrates excellent antibacterial performance and outperforms two commercial portable plasma jet devices (kINPen^®^ MED Plasma Jet and J-Plasma^®^) and a lab-based plasma jet system with respect to *in vitro* antimicrobial efficacy. Our mini plasma jet eradicated five different strains of bacteria in planktonic culture (*Staphylococcus aureus* ATCC 25923, ATCC 33592, ATCC BAA-1717, and EMRSA-16, as well as *Pseudomonas aeruginosa* PAO1) within 40 s, which is significantly shorter than the eradication times required by commercial and the lab-based plasma jets under the same conditions. In contrast, two commercial plasma jets could not disinfect effectively within 300 s and the lab-based system within 180 s. The test results show that our mini plasma jet can generate more reactive oxygen species (ROS, specifically H_2_O_2_) and reactive nitrogen species (RNS, specifically NO_3_^−^) than the other three systems.

## 1. Introduction

Plasma, recognised as the fourth state of matter alongside solid, liquid, and gas, is typically generated through gas discharge processes (Eliezer and Eliezer, 2001). Based on particle energy distribution and characteristics, plasma is classified into thermal (hot) and non-thermal (cold) plasma (Samal, 2017). In recent years, non-thermal plasma (NTP) has gained significant interest due to its extensive applicability within medical and biological fields (Moszczyńska et al., 2023; Zashev et al., 2020). Given its near-room-temperature operation, NTP is especially advantageous for processing heat-sensitive biomaterials without damaging their structural integrity (Mumtaz et al., 2023). Moreover, the application of NTP in biological research and clinical practice has led to the development of a new interdisciplinary field termed “Plasma Medicine” (Laroussi, 2018; Kong et al., 2009; Usta et al., 2019).

Biomedical applications of NTP span various fields including wound healing (Kang et al., 2017; Dubey et al., 2022), muscle repair and localized inflammatory responses (Smith et al., 2025), cancer therapy (Trimukhe et al., 2022), blood coagulation (Ke and Huang, 2016), dental treatments (Wu et al., 2015; Lata et al., 2022), microbial decontamination (Xu and Bassi, 2024), and water purification (Maybin et al., 2024; Wielogorska et al., 2023). Of these, antimicrobial applications have received significant attention. NTP has been extensively utilized in medical and hygiene related fields. For example, plasma-based biomedical surface synthesis (Bekeschus et al., 2019), skin tissue treatment (Robert et al., 2025), and eradication of resistant pathogens on skin and wounds (Maho et al., 2021; Daeschlein et al., 2015) have all benefited from its application. The potent bactericidal activity of NTP is primarily due to the generation of reactive oxygen and nitrogen species, resulting in oxidative stress and microbial death, even within tolerant biofilm communities (Maybin et al., 2023; Alshraiedeh et al., 2020; Shambharkar et al., 2024). Several challenges must be overcome before innovative NTP technologies can be used for practical applications. These challenges include plasma power source design, lack of real-time diagnostics, modeling and simulation, and understanding of the underlying principles across different applications for further enhancement of plasma performance (Lu et al., 2022). Addressing these requires collaboration across multidisciplinary communities. A recent publication (Thompson et al., 2025) highlighted the diversity in plasma types and devices employed, underscoring the importance of device design and optimization for maximizing antimicrobial efficiency and tissue compatibility.

Atmospheric pressure plasma jet (APPJ) systems, a subset of NTP devices, are particularly favoured for antimicrobial applications due to their straightforward construction, operational simplicity, and adaptability to various environmental conditions (Lackmann and Bandow, 2014; Do Nascimento et al., 2023; Das et al., 2024). While numerous studies have demonstrated excellent bactericidal outcomes using APPJ and elucidated underlying antimicrobial mechanisms (Daeschlein et al., 2012; Lackmann and Bandow, 2014; Judée et al., 2014; Lehmann et al., 2017; Guanlei et al., 2018; Nasser et al., 2022; Das et al., 2023; Do Nascimento et al., 2023), existing designs typically emphasize modifications to plasma tube geometries and electrode configurations. In contrast, limited attention has been dedicated to optimizing APPJ power supply systems, with many studies relying on bulky commercial power supply units that require mains electricity, significantly restricting their portability and versatility.

A portable and miniaturized power supply design can make the application of APPJ more convenient and expand the use cases. In 2019, Usta et al. reported a prototype design of a portable and battery-powered NTP device and achieved good antibacterial results for *Escherichia coli* and *Staphylococcus aureus* (Usta et al., 2019). Their work demonstrated the feasibility of a portable power supply design for NTP.

In this study, we introduce a miniaturized APPJ device driven by a custom-designed, lightweight (~100 g), and compact (15 cm × 10 cm × 2 cm) power supply (Length × Width × Height). To evaluate antimicrobial performance, we tested the system against two common clinical pathogens: *S. aureus* and *Pseudomonas aeruginosa*, both of which frequently cause severe infections (Serra et al., 2015; Sabat et al., 2021; Nguyen and Oglesby-Sherrouse, 2016). Our miniaturized APPJ successfully sterilized bacteria completely within just 40 s under the specified test conditions, significantly outperforming two commercial devices (kINPen^®^ MED Plasma Jet and J-Plasma^®^) and a larger previously characterized in-house kHz-driven plasma jet system (neoplas med GmbH, 2025; Apyx Medical, 2025; Alkawareek et al., 2012). The test results also showed that our mini plasma system generates more nitrate and hydrogen peroxide within the same time compared to the three APPJs. These test results demonstrated the excellent antimicrobial ability of our mini APPJ.

## 2. Experimental

### 2.1. Mini Atmospheric Pressure Plasma Jet (APPJ) device design and fabrication

**Fig. 1.**
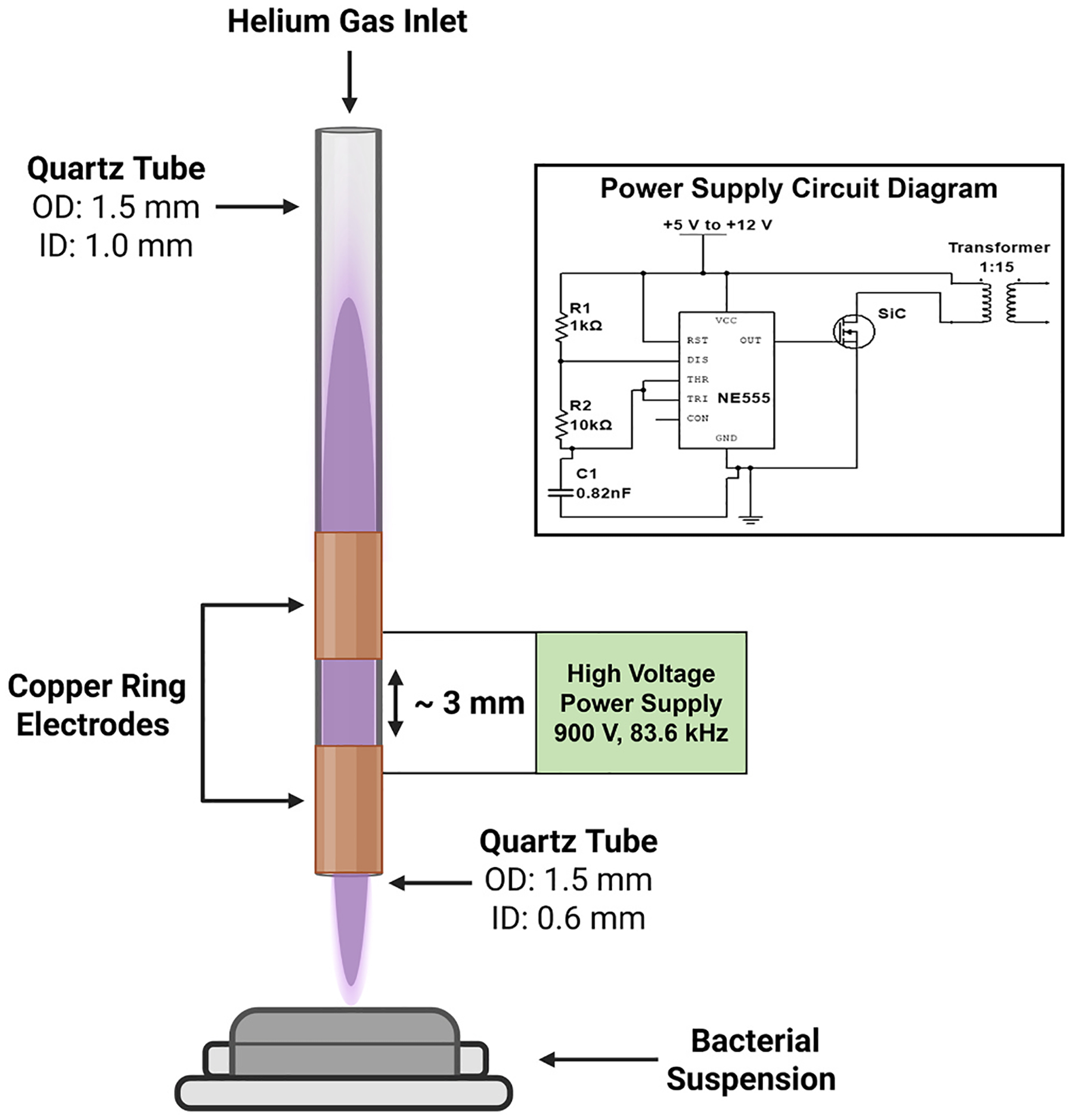
Schematic of the mini plasma jet device and test setup. The plasma jet generation is powered by a custom-made mini power supply unit. Created in BioRender. McClenaghan, L. (2026) https://BioRender.com/u5de2g6.

The schematic of the APPJ device used in this study is depicted in Fig. 1. The APPJ power supply used in this study is the same as our previously published work related to chemical sensing (Mao et al., 2023, 2024). The power supply design presented by Li et al. (Li et al., 2021) was modified to generate a helium dielectric barrier discharge plasma. The long-term thermal stability of the modified power supply was greatly improved by using a silicon carbide transistor (from ~15 min to more than 20 h of continuous operation). Notably, the current design still allows for further miniaturization. The current power supply is capable of producing an output AC signal of ~900 V_RMS_ at ~83.6 kHz frequency with a DC input of 10 V and 0.5 A. Considering that the output voltage of our power supply is relatively low compared to the published work (several to tens of kV (Daeschlein et al., 2012; Guanlei et al., 2018; Shi et al., 2022; Do Nascimento et al., 2023; Kanu et al., 2024)), we changed the design of glass tube to obtain an increased visible plume intensity. First, we used the plasma glass tube with a small inner diameter (ID) and limited the tube length based on the initial test results. The APPJ plasma glass tube with a total length of ~54 mm is custom made from laboratory glass and uses helium as the working gas. The glass tube ID at the inlet is 1.0 mm and the outer diameter (OD) is 1.5 mm. A thinner walled glass tube allows more efficient transmission of the applied electric field from the outer electrodes into the feed gas, thereby increasing the electric field strength inside the tube and promoting the formation of a more intense plasma plume. Some studies suggest that a moderate increase in the gas flow rate at the outlet can increase the plasma jet length when helium flow is laminar (Shi et al., 2022; Nasser et al., 2022). However, as the flow rate increases, the helium flow will transition from laminar to turbulent, hindering the formation of a plasma jet (Robert et al., 2014; Ghasemi et al., 2013; Park et al., 2010). In this work, the helium flow rate was fixed at 2 L/minute (~£0.2/minute). We reduced the outlet diameter of the glass tube to ~0.6 mm to increase the helium velocity and concentrate the gas flow. This design resulted in a longer plasma jet compared to the unmodified glass tube with an outlet diameter of 1 mm. Moreover, we also optimized the length of the copper ring electrodes (5 mm) using simulations. Several published studies have indicated that the electrode position and the distance between electrodes have a significant effect on APPJ generation (Jiang et al., 2009; Zhang et al., 2017; Veeraiah et al., 2019; Wang et al., 2019). In addition, for the APPJ with a ring-ring electrode configuration, a smaller electrode distance is preferred to increase axial electric field intensity at the centre of the tube and it also makes the formation of the plasma jet easier (Wang et al., 2024). However, a very small distance between the electrodes can result in air breakdown between the electrode edges. After performing several sets of experiments, we found that a more intense plasma plume can be safely created when the distance between the ring electrodes is ~3 mm.

The bacterial suspension sample is placed in position directly below the plasma jet and comes in direct contact with the APPJ during antibacterial tests. In fact, placing the electrode close to the outlet also helps to produce more reactive oxygen species (ROS) and reactive nitrogen species (RNS) when APPJ is in contact with the bacterial suspension since this design will increase the energy transfer from plasma to sample (Lietz and Kushner, 2018). The total component cost of our mini plasma jet system was less than U.S. $45 (Table S1). During tests, both the custom power supply and mini plasma jet demonstrated excellent operational stability in long-term tests and multiple treatment cycles. The custom power supply was in operation for over 20 h before conducting antibacterial testing. Additionally, we utilized the optical emission spectra (OES) of the plasma jet to characterize long-term stability and device-to-device variability (n = 5). A stable OES was captured by placing a mini spectrometer close to the plasma jet, clearly demonstrating that our device can operate continuously for at least eight hours and six days (Figure S1). Similarly, the distribution and intensity of optical emission peaks (Figure S2) show negligible variation across different devices. Moreover, we also evaluated the output voltage waveform using a high-voltage probe (Micsig DP10013 Differential Probe), and the measured waveform (Figure S3) was consistent with the transformer output voltage specified in the datasheet.

### 2.2. Mini plasma jet and antimicrobial test setup

**Fig. 2.**
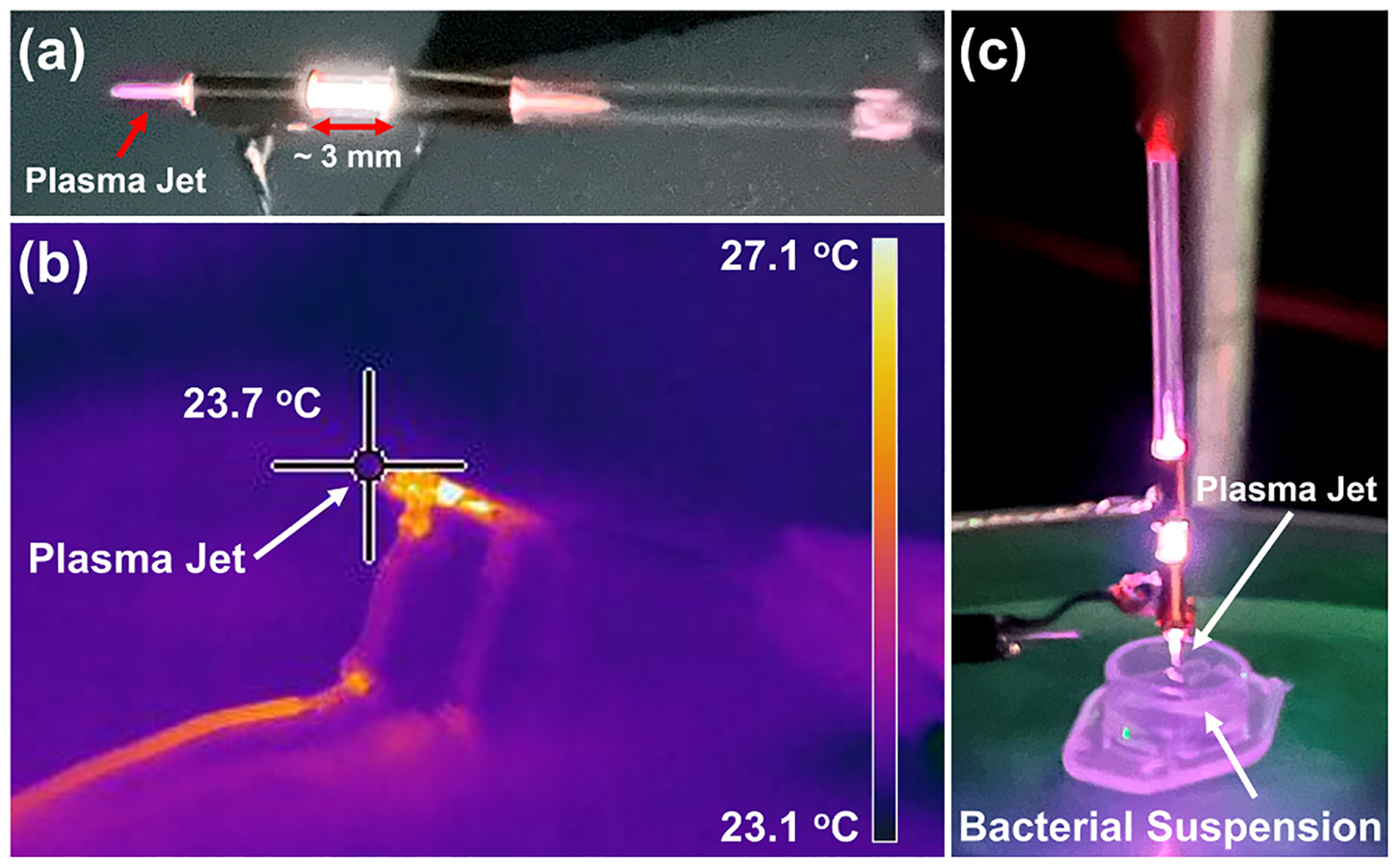
(a) Plasma jet generation after the device is turned on. (b) Thermal image of plasma jet during operation and the corresponding plasma jet temperature. (c) Plasma jet disinfection experimental setup, the liquid surface is positioned at a distance of ~5 mm from the bottom of the glass tube (image taken in a dark environment).

An image of the mini plasma jet in operation is shown in Fig. 2a. The plasma jet length is ~4 mm in open air and increases to ~5–8 mm after touching the liquid surface. Please note that the light spot (Fig. 2a) on the right side of glass tube is not plasma emission. The light spot is caused by the contact between photons emitted by the plasma and the silicone tubing. Additionally, plasma discharge is also generated at the top electrode inside the glass tube due to the symmetrical configuration of the ring electrodes. The device thermal image (taken by a FLIR SC640 thermal camera) shows that the temperature of mini plasma jet is ~23.7°C as indicated by the cursor (Fig. 2b). Due to the limited temporal and spatial resolution of our thermal camera, we were unable to measure the temperature distribution of mini plasma jet. Rouillard, et al. demonstrated that transient hot spots can be generated during plasma jet operation, while the overall jet temperature remains below 40 °C (Rouillard et al., 2024). Fig. 2c illustrates the setup used to evaluate the antimicrobial activity of this plasma jet. The helium gas exiting at high velocity disturbs the liquid surface and creates a small depression in the liquid, which increases the contact area between the plasma jet and liquid.

### 2.3. Bacterial strains and culture conditions

Five bacterial strains were used in this study, including *S. aureus* ATCC 25923, ATCC 33592, ATCC BAA-1717 (USA300, CA-MRSA), EMRSA-16, and *P. aeruginosa* PAO1. These strains were selected to represent clinically relevant Gram-positive and Gram-negative pathogens. All bacterial strains were maintained in Mueller-Hinton Broth (MHB) (Oxoid, UK) and incubated at 37°C under aerobic conditions. For planktonic culture experiments, overnight bacterial cultures were diluted in fresh MHB to an optical density at 600 nm (OD600) of 0.1, corresponding to approximately 10^7^ CFU/mL. These cultures were incubated at 37 °C with agitation at 100 rpm in an orbital shaker until reaching the mid-logarithmic growth phase. Biofilm formation was carried out using UV-sterilised titanium sheets (Grade 1, thickness: 1.0 mm, size: 5 mm × 5 mm, Goodfellow Cambridge Limited, UK). For biofilm development, overnight cultures of *S. aureus* ATCC 25923 were adjusted to an OD_600_ of 0.1 in fresh MHB. A total of 500 μL of the bacterial suspension was added to each well of a 24-well plate containing a single titanium sheet, and the plates were incubated statically at 37°C for 24 h. Following incubation, non-adherent planktonic cells were removed by gently rinsing the biofilms three times with 500 μL of phosphate-buffered saline (PBS). Biofilm growth conditions were standardized across experiments, and baseline (time 0) CFU were quantified prior to plasma exposure (reported in Fig. 7e–f).

### 2.4. Plasma treatment of planktonic and biofilm cultures on titanium substrates

Planktonic bacterial suspensions and biofilms grown on titanium substrates were exposed to plasma treatment using five distinct plasma devices: the novel Mini Jet described herein, kINPen Med (neoplas med GmbH, Greifswald, Germany), J-Plasma (Apyx^™^ Medical Corporation, Clearwater FL, USA), and our previously described in-house lab-based kHz (He), and kHz (He/O_2_) plasma jets (Alkawareek et al., 2012). The operating parameters of these five devices used in the experiments are summarized in Table S2. All devices were operated at matched stand-off distance and flow where feasible, and otherwise at manufacturer-recommended or device-constrained settings. Plasma was applied at a standardized distance of 10 mm (~5 mm for mini plasma jet) between the electrode/base of tube and the surface of the liquid bacterial cultures to ensure uniformity of treatment. The shorter treatment distance set for the mini plasma jet is due to its relatively short plume length and low applied voltage (~900 V_RMS_). A distance of 10 mm makes it difficult for the mini plasma jet to establish effective contact with the liquid surface. Similarly, the working distance for J-plasma was set to 6 mm. For biofilm treatments, the plasma device was continuously moved across the surface of the titanium sheets to minimize localized heating and provide uniform exposure across the treatment area. It should be noted that the scanning operation is necessary due to the small plasma spot size of our mini plasma jet, which represents a limitation of the current design. To achieve complete treatment, the titanium sheets were flipped midway through the plasma exposure process, allowing both sides to be exposed. Planktonic bacteria were subjected to plasma treatment at varying exposure times depending on the strain. *S. aureus* ATCC 25923 and *P. aeruginosa* PAO1 were treated for 0, 10, 20, 30, 40, 50, and 60 s. In contrast, *S. aureus* ATCC 33592, ATCC BAA-1717 (USA300), and EMRSA-16 were treated for longer durations of 0, 30, 60, 90, 120, 150, 180, 210, 240, 270, and 300 s. For biofilm cultures, *S. aureus* ATCC 25923 biofilms were treated for 0, 15, 30, 60, 120, and 300 s. Immediately after plasma exposure, biofilms were transferred into 500 μL of PBS in a 24-well plate and subjected to sonication for 20 min in an Elmasonic S30 ultrasonic bath (Elma Schmidbauer GmbH, Germany) at a frequency of 37 kHz. This process ensured the detachment and dispersal of biofilm-associated bacteria for further analysis. Bacterial suspensions were serially diluted and plated onto Mueller-Hinton Agar (MHA) and enumerated using the Miles and Misra method to determine bacterial survival rates.

### 2.5. Quantification of reactive oxygen and nitrogen species

The concentrations of key reactive oxygen and nitrogen species, including hydrogen peroxide (H_2_O_2_), nitrate (NO_3_^−^), and nitrite (NO_2_^−^) in plasma-treated samples suspended in PBS and deionized water were quantified using commercially available colorimetric assay kits as described below. The H_2_O_2_ levels were quantified using the Amplex^®^ Red Hydrogen Peroxide/Peroxidase Assay Kit (ThermoFisher, UK) following the manufacturer’s protocol. Plasma-treated samples were diluted in 1 × reaction buffer, transferred to a 96-well plate, and incubated with Amplex Red Working Solution for 30 min at room temperature. Absorbance was recorded at 560 nm using a FLUOstar^®^ Omega Multi-mode microplate reader (BMG Labtech, UK), and H_2_O_2_ concentration was determined against a standard curve. The nitrate concentration was measured using a nitrate assay kit (Sigma-Aldrich). 80 μL of the NO_3_^−^ solution was added to each well, followed by 10 μL of plasma-treated sample and 10 μL of the reaction buffer. After a 10-minute incubation, absorbance was recorded at 340 nm. Nitrite concentration was determined using the Griess Reagent Kit (Sigma Aldrich), where 50 μL of plasma-treated PBS was combined with 50 μL of pre-mixed Griess reagent, incubated for 30 min in the dark, and absorbance was measured at 548 nm as described previously (Shambharkar et al., 2024). Given the complexity of plasma-liquid chemistry, these colorimetric assays are interpreted as operational readouts of stable end-products (assay-equivalents) under our conditions and may not capture all short-lived species.

### 2.6. pH measurements

To assess the effect of plasma treatment on pH, measurements were taken immediately following plasma exposure using a calibrated Hanna Edge pH meter. Readings were recorded in triplicate for both PBS and deionized water to evaluate the impact of plasma-generated reactive species on solution acidity. The pH remained stable in PBS-treated samples, whereas a significant pH drop was observed in plasma-treated deionized water.

### 2.7. Scavenger assays

To assess the contribution of specific reactive species to plasma-mediated bacterial inactivation, scavenger assays were conducted based on previous studies, the following scavengers and concentrations were used: sodium pyruvate (10 mM) as a scavenger of hydrogen peroxide (Zhang et al., 2021), tiron (20 mM) as a scavenger of superoxide (O_2_^−^•) (Guo et al., 2018), and L-histidine (10 mM) as a scavenger of singlet oxygen (^1^O_2_) and other ROS and haemoglobin (20 μM) as a scavenger for nitric oxide (Zhou et al., 2019). The effect of scavenger addition on bacterial survival was assessed through CFU enumeration.

### 2.8. Comparative NTP devices and operating parameters

To contextualise the performance of the mini plasma jet, four additional plasma sources were assessed under matched experimental conditions. The kINPen^®^ MED (neoplas tools GmbH) was operated with a 4 L/min argon flow at a fixed 10 mm distance. The J-Plasma^®^ Precise Open Handpiece (Apyx Medical) was operated at 6 W (70% output) with 4 L/min gas flow, held at ~6 mm and moved at ~1 cm/s, as previously described (Thompson et al., 2025). An in-house kHz-driven dielectric barrier discharge jets were operated with either pure helium or a 0.5% helium-oxygen admixture, both at 2 L/min and 6 kV, 20 kHz AC power. Each was configured with 25 mm electrode spacing and a 10 mm treatment distance and has been extensively characterised (Maybin et al., 2023; Flynn et al., 2015; Alkawareek et al., 2014b, 2012; Alshraiedeh et al., 2016; Barakat et al., 2019; Mcdonnell et al., 2022). All planktonic cultures and titanium-supported biofilms were exposed to plasma generated by each device for durations ranging from 10 to 300 s. Post-treatment, samples were processed for viability assays and reactive species quantification as described above. Comparative ROS/RNS generation was evaluated in deionised water following 60 s exposure per device.

### 2.9. Statistical analysis

All experiments were conducted in triplicate using independently grown cultures. Data were analysed using GraphPad Prism (version 10.4.1, GraphPad Software, San Diego, CA, USA). Statistical significance was assessed using one-way ANOVA with Tukey’s post-hoc test for multiple comparisons. A p-value of less than 0.05 was considered statistically significant.

## 3. Results and discussion

### 3.1. Simulation of electric field distribution

**Fig. 3.**
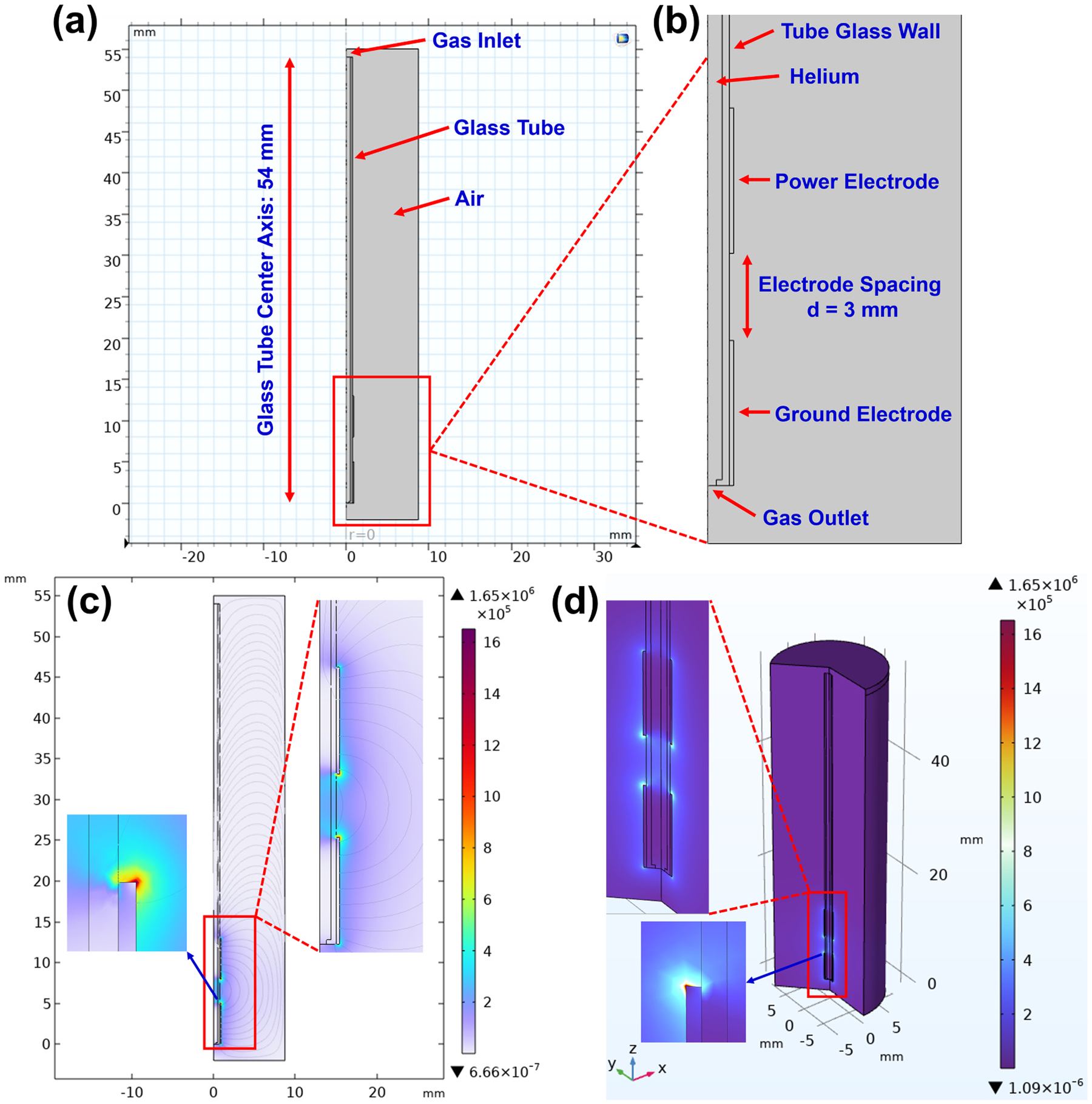
(a) 2D axis-symmetric model of mini plasma jet tube (d = 3 mm). (b) Zoomed-in image of electrodes and gas outlet area. (c) Simulation of 2D axisymmetric electric field distribution of mini plasma jet glass tube and zoomed-in view of the electrodes. (d) Spatial distribution of electric field in 3D based on 2D simulation results and zoomed-in view of the electrodes. Note that the electric field intensity is close to 0 in the areas away from the top ring electrode.

The electric field intensity and distribution inside the glass tube are critical for a stable plasma jet generation. For ring-ring type APPJ device, the electrode thickness, length, and the distance between the ring electrodes all have an impact on the electric field distribution inside the glass tube (Wang et al., 2024, 2022). In this work, the mini plasma jet ring electrode dimensions are fixed. Therefore, we simulated the electric field distribution at different electrode spacings using a 2D axis-symmetric model (COMSOL Multiphysics). As shown in Fig. 3a, we built the 2D axis-symmetric model based on the actual device dimensions. In this model, the geometric symmetry axis is the central axis of the glass tube. Fig. 3b shows a zoomed-in image of the modelled area including copper ring electrodes and tube gas outlet. The inside of the glass tube is set as helium filled, and the outside is surrounded by air. The top ring electrode is set as the power electrode, and the bottom electrode is set as the ground electrode. The voltage applied on the power electrode is set to 900 V_RMS_, which is the output voltage value of our power supply. To model the ground electrode, its position is fixed and set at the bottom edge of the tube outlet. The electrode spacing is represented by the parameter d in this study (d = 3 mm is used for model demonstration). We simulated the glass tube electric field distribution by changing the value of d from 1 mm to 20 mm with a step size of 1 mm. Fig. 3c shows the 2D axis-symmetric electric field spatial distribution of a mini plasma jet when the electrode spacing is 3 mm. Additionally, the 3D electric field intensity distribution of the tube and the surrounding space is also plotted based on the 2D simulation results (Fig. 3d). The simulation clearly shows that the high intensity electric field is mainly distributed in the areas near the two copper ring electrodes, and the electric field intensity is very low (close to 0) in the areas far away from the top ring electrode.

**Fig. 4.**
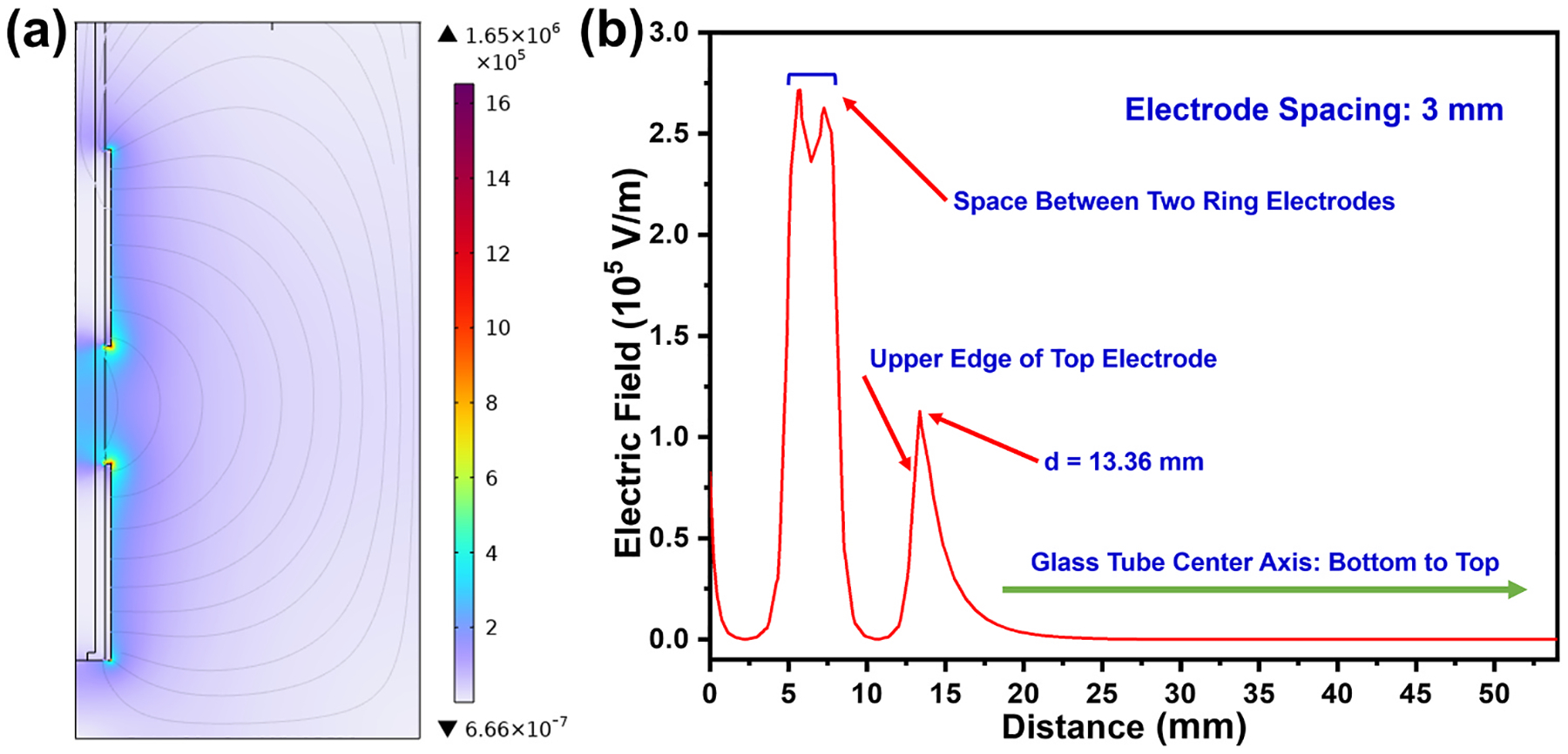
(a) Spatial distribution of electric field strength corresponding to the same area as in Fig. 3b. (b) Electric field intensity distribution along the central axis of mini plasma jet glass tube from bottom to the top when spacing between the two copper ring electrodes is 3 mm.

Fig. 4a shows the results of electric field distribution (same area as Fig. 3b) near the ring electrodes. The maximum electric field strength is located at the bottom edge of the top electrode and the top edge of the bottom electrode. The minimum electric field intensity is distributed in the inside space of the glass tube area covered by the copper ring electrodes. For the space around the electrode, its field intensity gradually decreases as the distance from the electrode edge increases. Fig. 4b presents the electric field intensity distribution inside the glass tube along the central axis (from bottom to top). The maximum axial electric field intensity is located at the position between the two ring electrodes. After reaching a peak value near the electrode edge, the field intensity begins to decrease as we move towards the centre position between the two electrodes and reaches a peak value again near the edge of the top electrode. In addition, the electric field strength changes significantly around the electrode edges. For example, the field intensity reaches a peak in the position slightly outside of the top electrode edge, but the field intensity on both sides decreases rapidly (Fig. 4b). The field intensity is also very low around the axial positions away from the top electrode edge.

**Fig. 5.**
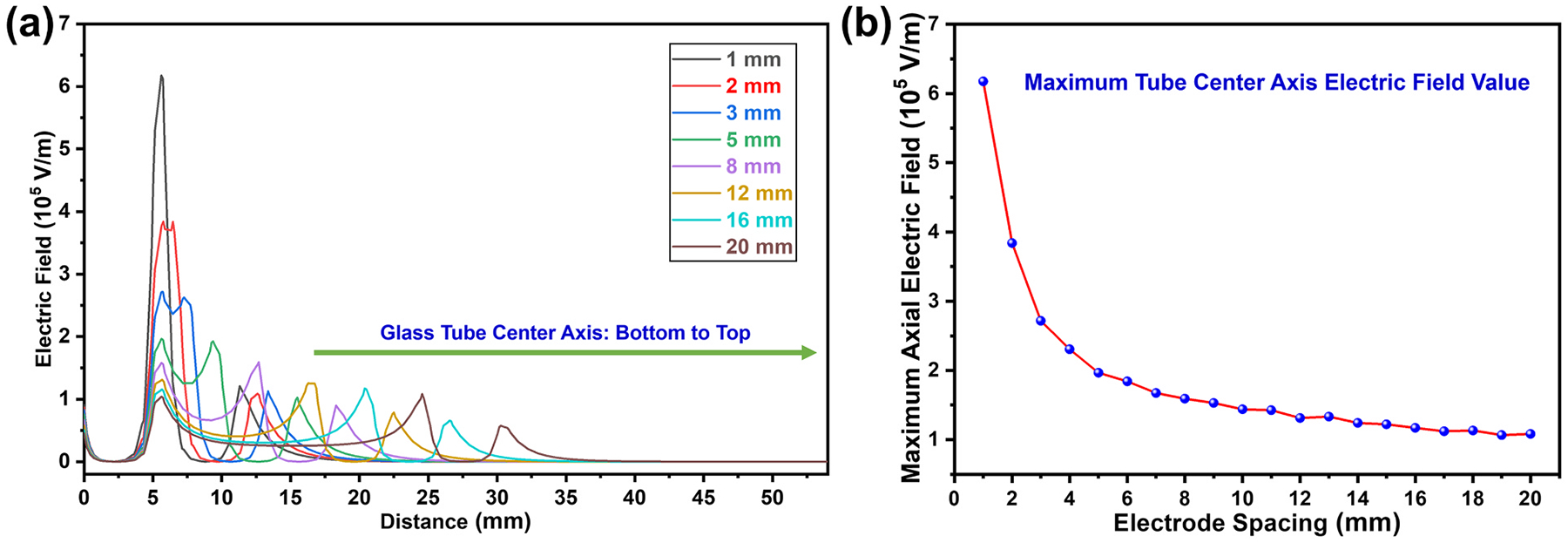
(a) Electric field intensity distribution corresponding to central axis of the glass tube at electrode spacings of 1, 2, 3, 5, 8, 12, 16, and 20 mm. (b) The maximum electric field intensity along the mini plasma jet glass tube central axis at electrode spacings from 1 to 20 mm.

Fig. 5 shows axial electric field intensity distribution at the centre of tube (Fig. 5a) and the maximum axial electric field intensity value at different electrode spacings (Fig. 5b). It can be clearly seen that the overall axial electric field intensity decreases as the electrode spacing increases from 1 to 20 mm. The decrease in electric field is most obvious (Fig. 5a and b) when the electrode spacing increases from 1 to 5 mm. As shown in Fig. 5b, the maximum axial field strength decreased from 6.18 × 10^5^ V/m to 1.97 × 10^5^ V/m. From 5 mm to 20 mm, the maximum axial electric field intensity value gradually decreases to 1.08 × 10^5^ V/m. Although the intensity values are different, the axial electric field distributions at different electrode spacings are still similar to the distribution in Fig. 4b. The high intensity electric field regions are distributed near the axial positions corresponding to the copper ring electrode edges.

A higher electric field strength facilitates ionization and promotes plasma jet formation. However, when the electrode spacing was less than 3 mm, electrical breakdown of air was frequently observed, typically near the electrode edges where local field strengths peaked (Fig. 4a). Conversely, electrode spacings greater than 5 mm failed to produce a visible plasma jet. Based on this, a 3 mm spacing was selected as optimal.

The plasma jet electric field is the combination of the electrostatic (Laplacian) electric field caused by the applied potential and the space charge electric field generated by the plasma (Naidis and Walsh, 2013; Wang et al., 2022; Viegas et al., 2022). Helium flow was not included in the simulation. This simplification may affect the actual spatial distribution of the electric field (Liu et al., 2023). The absence of these conditions will cause differences between our simulation results and the actual spatial electric field distribution when mini plasma jet is generated. However, the purpose of our simulation is to qualitatively study the effect of electrode spacing on spatial electric field distribution, especially the effect on maximum axial electric field intensity. Although model conditions are simplified, our simulation still achieved similar results compared to the studies that include the plasma generation process (Zhu et al., 2018; Wang et al., 2023). More importantly, our electric field simulation results are of the same order of magnitude (10^5^ V/m) as mentioned in the published work based on experimental measurements (Robert et al., 2015; Sretenović et al., 2017; Orr et al., 2020). Therefore, our simulation results can provide an excellent reference for the ring electrode position design. An optimized electrode configuration is critical for plasma jet generation since our plasma power voltage is relatively low (900 V_RMS_). In fact, it was difficult for our device to generate plasma jet due to the low voltage. The tube geometry design modification (narrow outlet) and electrode position optimization are critical for the generation of a stable plasma plume.

### 3.2. Physicochemical characterisation of mini plasma jet

**Fig. 6.**
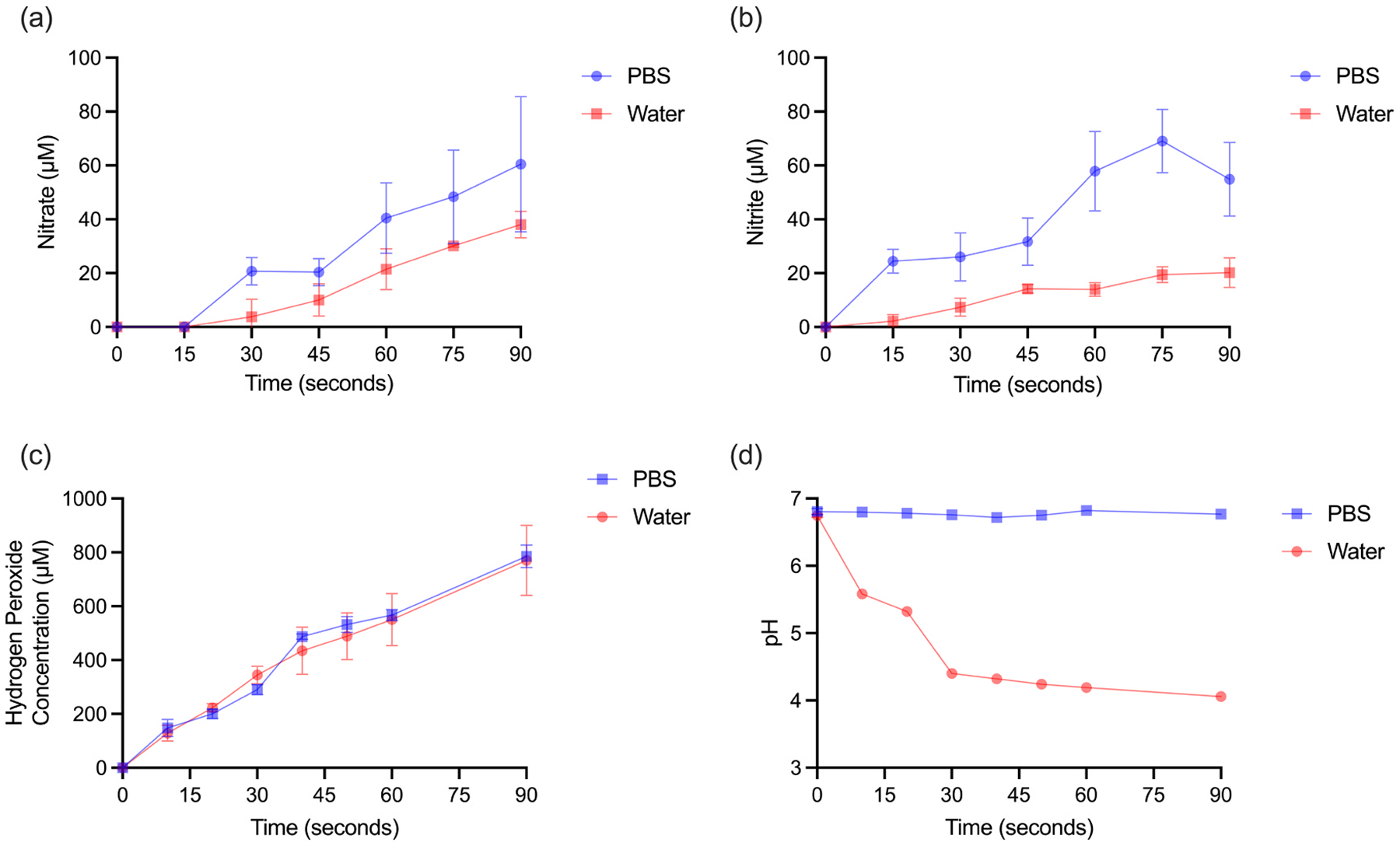
Quantification of ROS/RNS and pH changes in deionised water and PBS following plasma treatment. ${\text{NO}}_{3}^{-}$ (a), ${\text{NO}}_{2}^{-}$ (b), H_2_O_2_ (c), and pH (d) were measured in water or PBS exposed to a helium plasma jet over 90 s. The error bars represent the mean ± standard deviation from three biological replicates. (n = 3).

Over a 90-second exposure period, the mini plasma jet induced a marked accumulation of NO_3_^−^, NO_2_^−^, and H_2_O_2_ in both PBS and deionised water, though the dynamics differed between the two solutions (Fig. 6a–c). Concentrations of NO_3_^−^ in PBS reached up to ~90 μM by 90 s, while deionised water showed a slower but consistent increase, peaking around ~41 μM. Accumulation of NO_2_^−^ was more rapid in PBS, rising to ~81 μM at 75 s, with water plateauing at significantly lower levels (~27 μM). These differences likely reflect the greater buffering and ionic strength of PBS, which stabilises intermediate RNS and reduces their decomposition. The concentration of H_2_O_2_ increased almost linearly in both solutions, reaching concentrations of ~870 μM in water and ~780 μM in PBS by 90 s (Fig. 6c). Accompanying these ROS/RNS changes, pH in water dropped sharply from ~6.75 tô4.06, indicating acidification via plasma-generated acidic species such as nitric acid (HNO_3_) and nitrous acid (HNO_2_) (Fig. 6d). In contrast, PBS retained a near-neutral pH throughout, confirming its effective buffering capacity. These findings indicate that the mini plasma jet can generate physiologically relevant concentrations of ROS and RNS (e.g., >800 μM H_2_O_2_), which are well within the ranges known to bring about bactericidal and antibiofilm activity, despite operating at significantly lower voltages. The combination of high ROS/RNS generation and minimal temperature rise (up tô23.7°C) reinforces the potential of this low-cost and portable device for biological and clinical applications.

### 3.3. Antimicrobial susceptibility

**Fig. 7.**
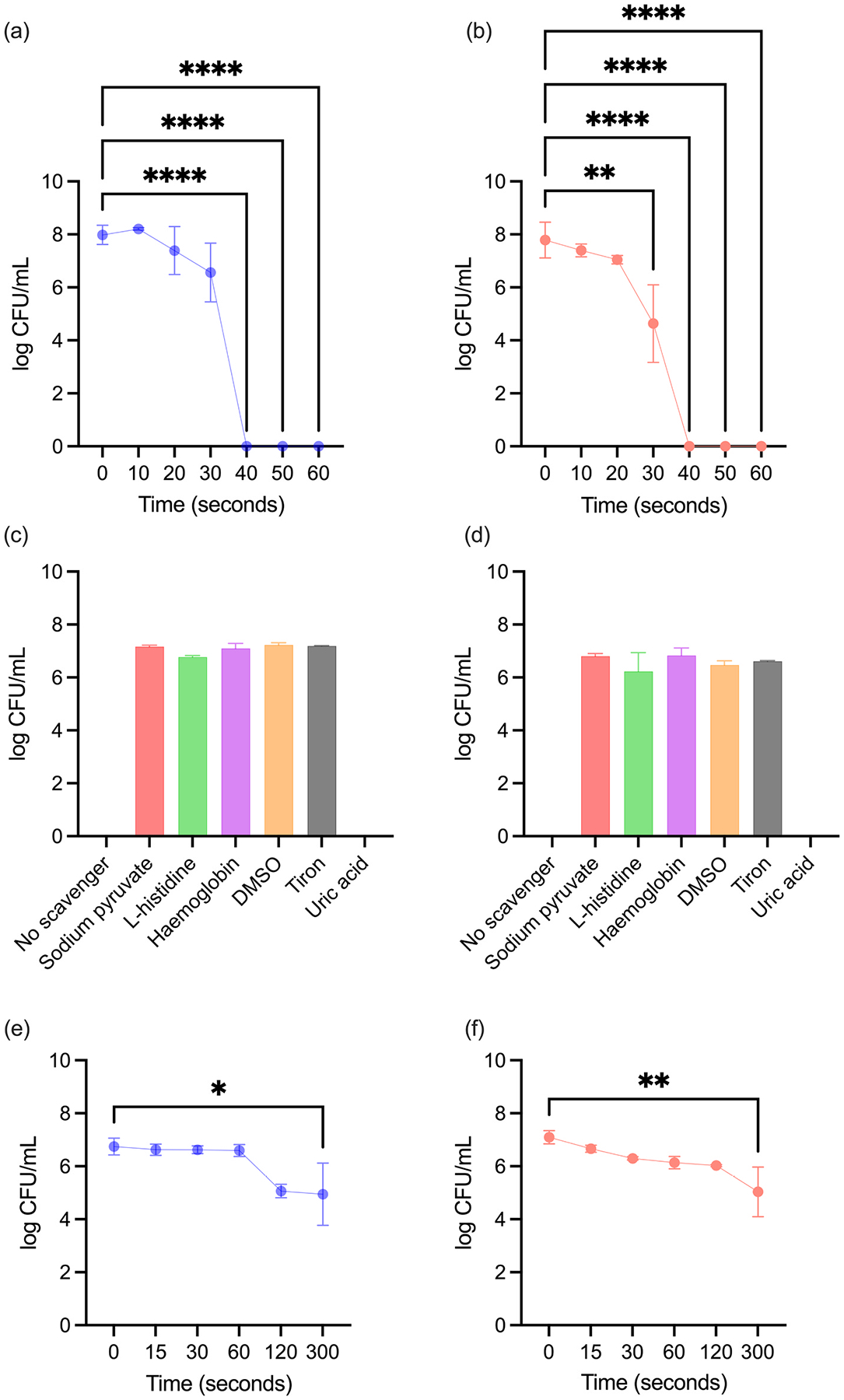
Antimicrobial activity of NTP against *S. aureus* and *P. aeruginosa* in planktonic and biofilm phenotypes. Log CFU/mL of planktonic *S. aureus* ATCC 25923 (a) and *P. aeruginosa* PAO1 (b) after plasma treatment. (c-d) Effects of reactive species scavengers on survival of *S. aureus* (c) and *P. aeruginosa* (d) following treatment. (e-f) Biofilm inactivation profiles of *S. aureus* (e) and *P. aeruginosa* (f) over extended plasma exposure. Asterisks indicate statistically significant differences compared to baseline (time = 0), * (p < 0.05), ** (p < 0.01), *** (p < 0.001), and **** (p < 0.0001), calculated using one-way ANOVA with Dunnett’s post-test. (n = 3).

The mini plasma jet achieved a > 6-log reduction in bacteria viability within 40 s against both Gram-positive *S. aureus* and Gram-negative *P. aeruginosa* in planktonic cultures (Fig. 7a–b). For *S. aureus* ATCC 25923, complete bacterial eradication was achieved within 40 s, with log_10_ CFU counts falling from ~8.2 initial bioburden to below detectable viability. Similarly, *P. aeruginosa* PAO1 time-kill kinetics showed a rapid and significant decline, with complete eradication by 40 s. These rapid reductions coincided with early accumulation of ROS/RNS, including NO_3_^−^, NO_2_^−^, and H_2_O_2_, implicating oxidative stress as a primary killing mechanism.

Scavenger assays were used as mechanistic probes to test whether quenching specific classes of ROS/RNS could partially rescue bacterial viability following plasma exposure (Fig. 7c–d), consistent with the approach used previously for plasma-activated liquids (Shambharkar et al., 2024; McClenaghan et al., 2025). In *S. aureus*, untreated plasma exposure resulted in CFU counts of ~6.8 log_10_. Sodium pyruvate (scavenges H_2_O_2_ and can intercept peroxynitrite-associated chemistry) and haemoglobin (peroxynitrite scavenger) each reduced plasma efficacy bŷ0.5–0.7 log_10_, consistent with a contribution from longer-lived oxidants. L-histidine (^1^O_2_ scavenger), DMSO (•OH scavenger), and Tiron (O_2_•^−^ scavenger) provided partial protection, supporting additional roles for short-lived radicals. The effect of radical scavengers on *P. aeruginosa* viability when exposed to plasma showed a similar profile (Fig. 7d). Uric acid, included as a broad antioxidant probe (used to test for non-specific ‘total oxidant’ rescue), did not provide measurable protection under our conditions.

Together, the pattern of partial rescue across multiple scavengers supports a multi-species oxidative mechanism with overlapping contributions, rather than allowing confident assignment of a single ‘primary effector’ from scavenger data alone. These observations are consistent with established plasma cytotoxicity models in which overlapping ROS/RNS impair membrane integrity, disrupt redox, and induce irreversible cell damage (Alkawareek et al., 2012, 2014a).

Beyond planktonic efficacy, the mini plasma jet exhibited substantial biofilm-inactivating potential (Fig. 7e–f). In *S. aureus* biofilms formed on titanium, viability remained stable up to 60 s but dropped by ~3-log_10_ between 120 and 300 s. *P. aeruginosa* biofilms declined more gradually, reaching ã2.5-log_10_ reduction by 300 s. These delayed effects reflect the physical and chemical resilience of biofilms, which limit ROS diffusion and shelter metabolically inactive cells. Nevertheless, the ability to reduce biofilm burden within 5 min supports the potential of this device as a surface-decontamination tool, especially relevant to persistent infections on implants, wounds, and medical equipment. These results align with studies showing that plasma treatment sensitizes biofilms by disrupting extracellular matrices and weakening redox defences (Maybin et al., 2023).

### 3.4. Comparison of activity against other NTP devices

To benchmark the performance of the mini plasma jet, we compared its antimicrobial activity and reactive species output with two commercial devices (kINPen^®^ MED, J-Plasma^®^) and two custom kHz-driven plasma jets using helium (He) or a helium–oxygen (He/O_2_) admixture.

**Fig. 8.**
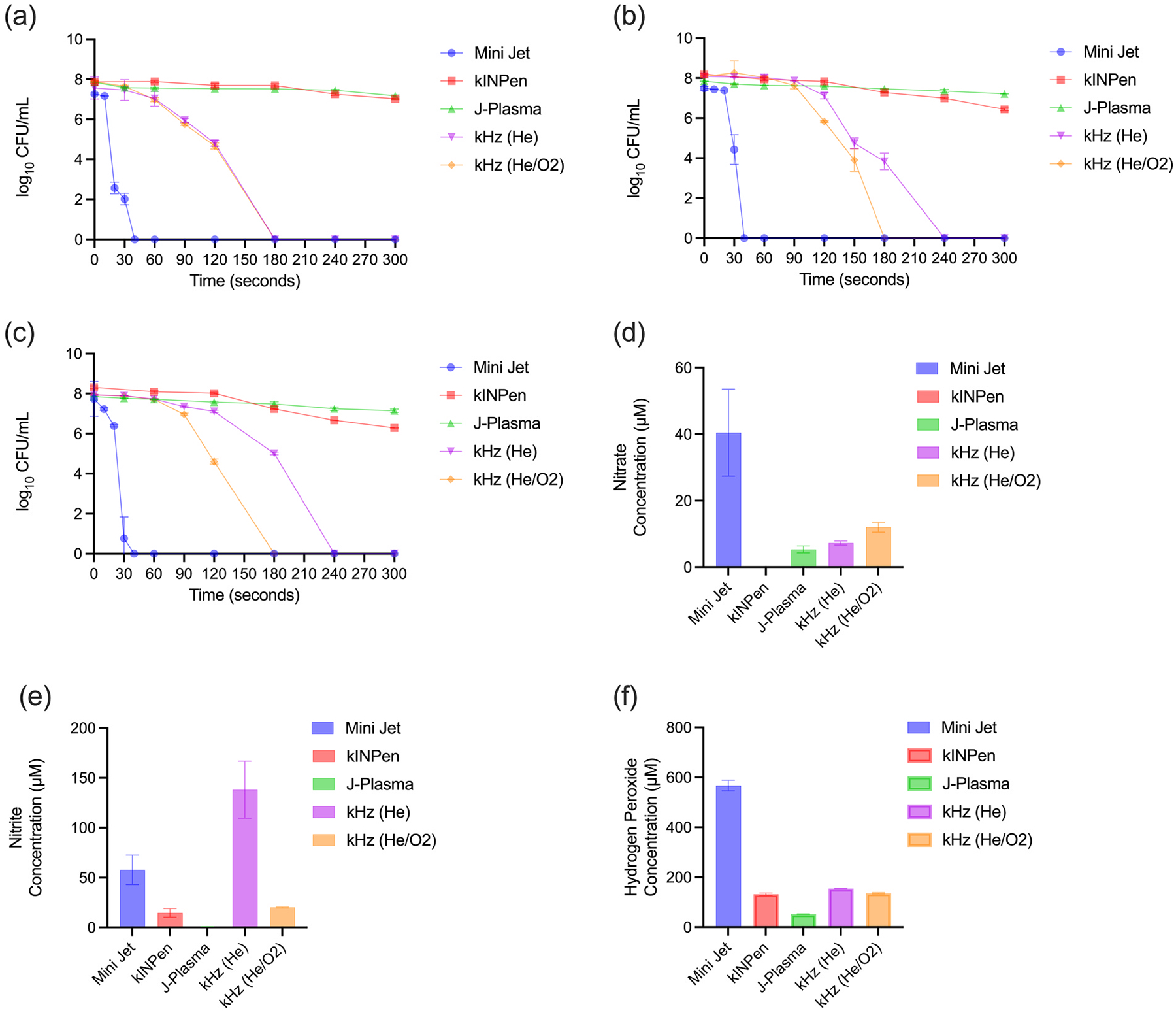
Comparative bactericidal activity of five plasma sources and corresponding ROS/RNS quantification. Time-kill curves showing the viability of planktonic (a) *S. aureus* EMRSA-16, (b) *S. aureus* ATCC 33592, and (c) *S. aureus* USA300 (ATCC BAA-1717) following treatment with five plasma devices: Mini Jet, kINPen, J-Plasma, and a custom-built kHz jet operating with He or He/O_2_. Concentrations of ${\text{NO}}_{3}^{-}$ (d), ${\text{NO}}_{2}^{-}$ (e), and H_2_O_2_ (f) were measured in PBS after 60 s of exposure from each plasma source. All data represent mean ± SD from three biological replicates (n = 3).

Across all three *S. aureus* strains tested, EMRSA-16, ATCC 33592, and USA300, the mini plasma jet achieved complete eradication within 40 s under the specified test conditions, significantly outperforming the kINPen^®^ MED and J-Plasma^®^, which produced minimal reductions (above 6.5–7.0 log_10_) even after 300 s of exposure. In contrast, the custom kHz plasma jets, particularly the He/O_2_ configuration, demonstrated delayed but complete inactivation of all strains by 180–240 s, confirming their efficacy at longer exposure times (Fig. 8a–c). Overall, the observed efficacy differences across devices are consistent with a combination of (i) higher measured ROS/RNS output (Fig. 8d–f) and (ii) differences in treatment geometry and dose delivery (stand-off distance, plume/spot size, and scanning requirements) under the conditions tested.

Interpretation of these benchmarking comparisons should consider treatment geometry. In our testing, comparator devices were operated at their stated working distances. However, the small treatment spot of the mini plasma jet necessitates scanning even for modest surface areas. Combined with the shorter stand-off distance requirement, this may limit throughput for large or uneven surfaces compared with broader-area sources. Similar constraints (small treatment area/uneven surface coverage) are widely recognized practical limitations for plasma-jet type sources. A further limitation is that long-duration performance drift and electrode wear for antibacterial testing were not quantified in the present study. Future work will include continuous-run stability testing, multi-cycle repeatability, and between-unit reproducibility for antibacterial measurements alongside reactive-species output.

These differences in bactericidal activity correlated closely with ROS/RNS concentrations measured in PBS after 60 s of plasma exposure (Fig. 8d–f). The mini jet produced the highest levels of H_2_O_2_ (~570–586 μM) and NO_3_^−^ (~52 μM)—both stable, membrane-permeable oxidants known to mediate oxidative killing. In contrast, the kINPen^®^ and J-Plasma^®^ produced lower H_2_O_2_ (~130 μM and ~50 μM, respectively) and negligible nitrate, likely contributing to their reduced efficacy.

Notably, the kHz (He) jet yielded the highest NO_2_^−^ levels (~150 μM) but was less effective at early time points, indicating that NO_2_^−^ alone is insufficient for rapid killing. These data suggest that bactericidal activity was most closely associated with higher measured H_2_O_2_ and NO_3_^−^ (assay-equivalents), whereas NO_2_^−^ alone was not associated with early killing.

This comparative analysis reinforces the critical role of device design, including electrode placement, outlet geometry, and power delivery, in shaping ROS/RNS output and antimicrobial efficacy, consistent with prior findings (Lietz and Kushner, 2018).

## 4. Conclusion

The mini plasma jet combines high antimicrobial efficacy, rapid action, and ultra-low power consumption (~5 W), making it well-suited for point-of-care applications in resource limited or mobile healthcare environments. Unlike conventional plasma systems that require bulky hardware and kV-range inputs, the mini jet’s compact, low-voltage design enables battery operation and integration into portable platforms/devices. These features enable practical application in wound care, implant sterilisation, and surface disinfection, where flexibility, safety, and ease of deployment are critical. This study shows that carefully engineered low-power plasma technologies can deliver bactericidal performance on par with, or better than, existing NTP devices, particularly in terms of ROS/RNS generation and time-kill kinetics. By combining low energy requirements with strong biological efficacy, the mini plasma jet represents a scalable and accessible solution for decentralised infection control in both clinical and field settings.

## Supplementary Material

1

## Data Availability

All data generated or analysed during this study are included in the results section of this paper.

## Appendix A. Supporting information

Supplementary data associated with this article can be found in the online version at doi:10.1016/j.eti.2026.104852.
