# Supplementary material for: A miniaturized and low-cost atmospheric pressure helium plasma jet device with high antimicrobial efficiency
Source: Environ Technol Innov. Author manuscript; Available in PMC 2026 Jun 24. (PMC13290277; doi:10.1016/j.eti.2026.104852)
Supplement: 1 [file NIHMS2187320-supplement-1.docx]

**Supporting Information**

**A Miniaturized and Low-Cost Atmospheric Pressure Helium Plasma Jet Device with High Antimicrobial Efficiency**

Jingqin Mao^a^, Thomas P. Thompson^b^, Laura A. McClenaghan^b^, Ross M. Duncan^b^, Brendan F. Gilmore^b^, and Hamza Shakeel^a*^

a. School of Electronics, Electrical Engineering and Computer Science, Queen’s University Belfast, Belfast, BT7 1NN, United Kingdom.

b. School of Pharmacy, Queen’s University Belfast, Belfast, BT7 1NN, United Kingdom.

* Corresponding Author: Hamza Shakeel. Email address: H.Shakeel@qub.ac.uk

**Table S1.** Cost of components used for manufacturing of custom power supply and plasma jet.

| **Custom Power Supply** | | **Plasma Jet Device** | |
| --- | --- | --- | --- |
| Component | Cost | Component | Cost |
| ZS1052(H) Trigger Coil ×1 | £2.29 | Glass Tube | <£0.3 |
| NE555P Timer ×1 | £0.38 | Copper Electrodes | <£0.2 |
| Type N SiC MOSFET ×1 | £20.24 | Silicone Tubing for Gas Delivery | ~£1 |
| Heatsink ×1 | £1.37 |  |  |
| Capacitor ×1 | ~£0.2 |  |  |
| 1k Ω Resistor ×1 | ~£0.1 |  |  |
| 10k Ω Variable Resistor ×1 | ~£2.30 |  |  |
| Jumper Wire | ~£1 |  |  |
| Stripboard | ~£0.5 |  |  |
|  | Total: ~£28.38 |  | Total: <£1.5 |
| Total Cost of Custom Power Supply and Plasma Jet Device: <£29.88 (~$41) | | | |

**Table S2.** Operating conditions used for antimicrobial benchmarking of the mini plasma jet and comparator plasma devices.

| **Device** | **Type** | **Gas** | **Flow Rate (L/min)** | **Power Settings** | **Frequency /Waveform** | **Stand-off Distance (mm)** | **Application Mode** |
| --- | --- | --- | --- | --- | --- | --- | --- |
| Mini plasma jet (custom) | Miniaturized APPJ (DBD) with external ring electrodes | Helium | 2 | ~5 W input (10 V, 0.5 A DC), output ~900 V peak | AC ~83.6 kHz (power supply output) | 5 | Static (planktonic); Scanned (biofilm) |
| kINPen MED (commercial) | Atmospheric-pressure plasma jet (medical device) | Argon | 4 | < 3.5 W | Sinusoidal AC ~1.0 ± 0.1 MHz, pulsed at 2.5 kHz (1:1 duty cycle) | 10 | Static (planktonic); Scanned (biofilm) |
| J-Plasma Precise Open Handpiece (commercial) | Helium plasma/RF surgical handpiece (Apyx Medical) | Helium | 4 | 6 W  (70% output) | RF generator | 6 | Static (planktonic); Scanned (biofilm) |
| kHz jet (He)  (custom) | In-house kHz-driven DBD jet | Helium | 2 | 6 kV (20 kHz AC power) | 20 kHz AC,  6 kV | 10 | Static (both) |
| kHz jet (He/O_2_)  (custom) | In-house kHz-driven DBD jet | Helium with 0.5% O_2_ | 2 | 6 kV (20 kHz AC power) | 20 kHz AC,  6 kV | 10 | Static (both) |


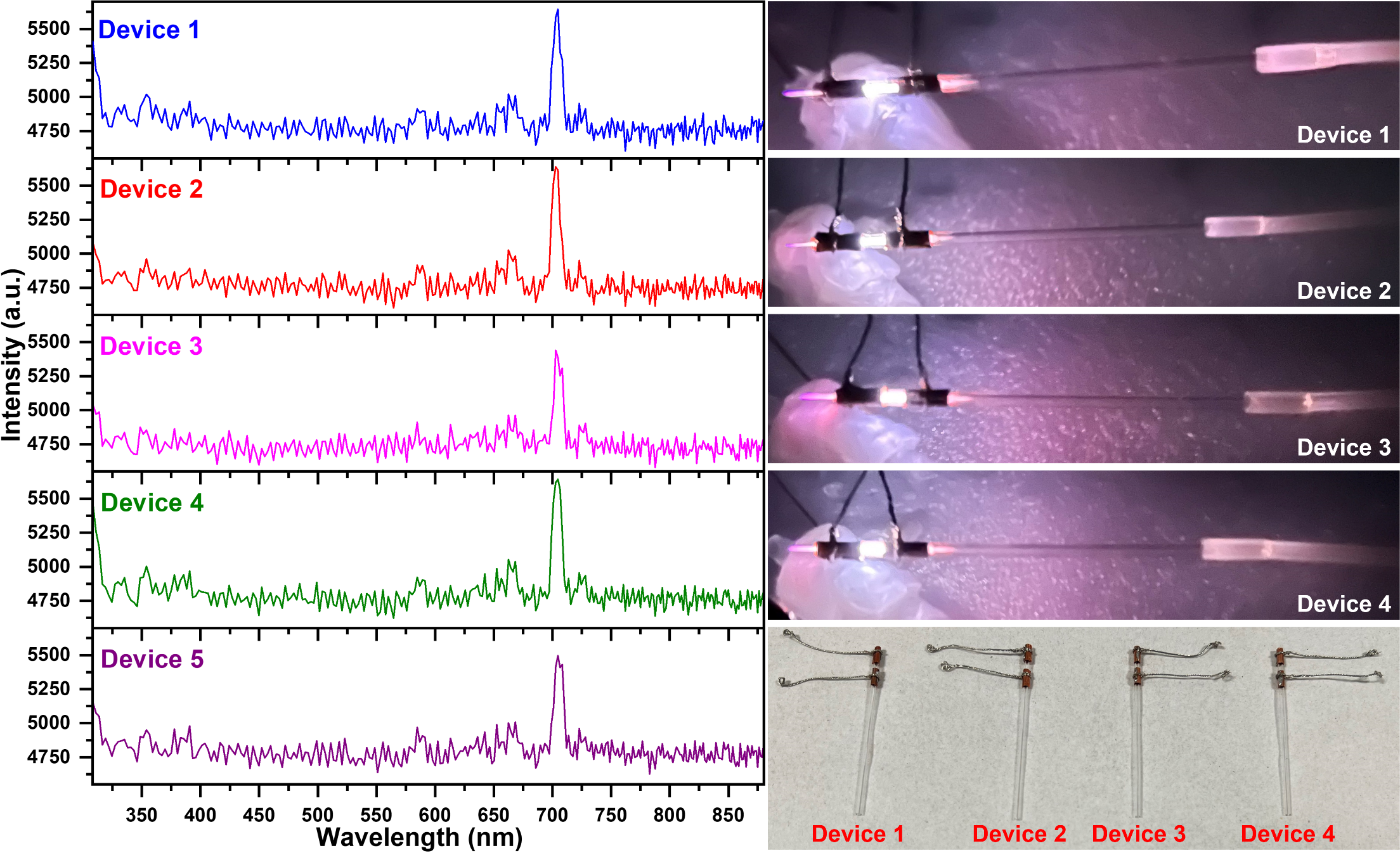


**Figure S1.** Images of different plasma jets and the corresponding optical emission spectra (OES) using a Mini spectrometer (Hamamatsu C12880MA). Device 5 is the one used in the antibacterial testing. It is easy to see that the OES of plasma jets generated by new devices 1-4 and device 5 have the same emission peak distribution and peak intensities.


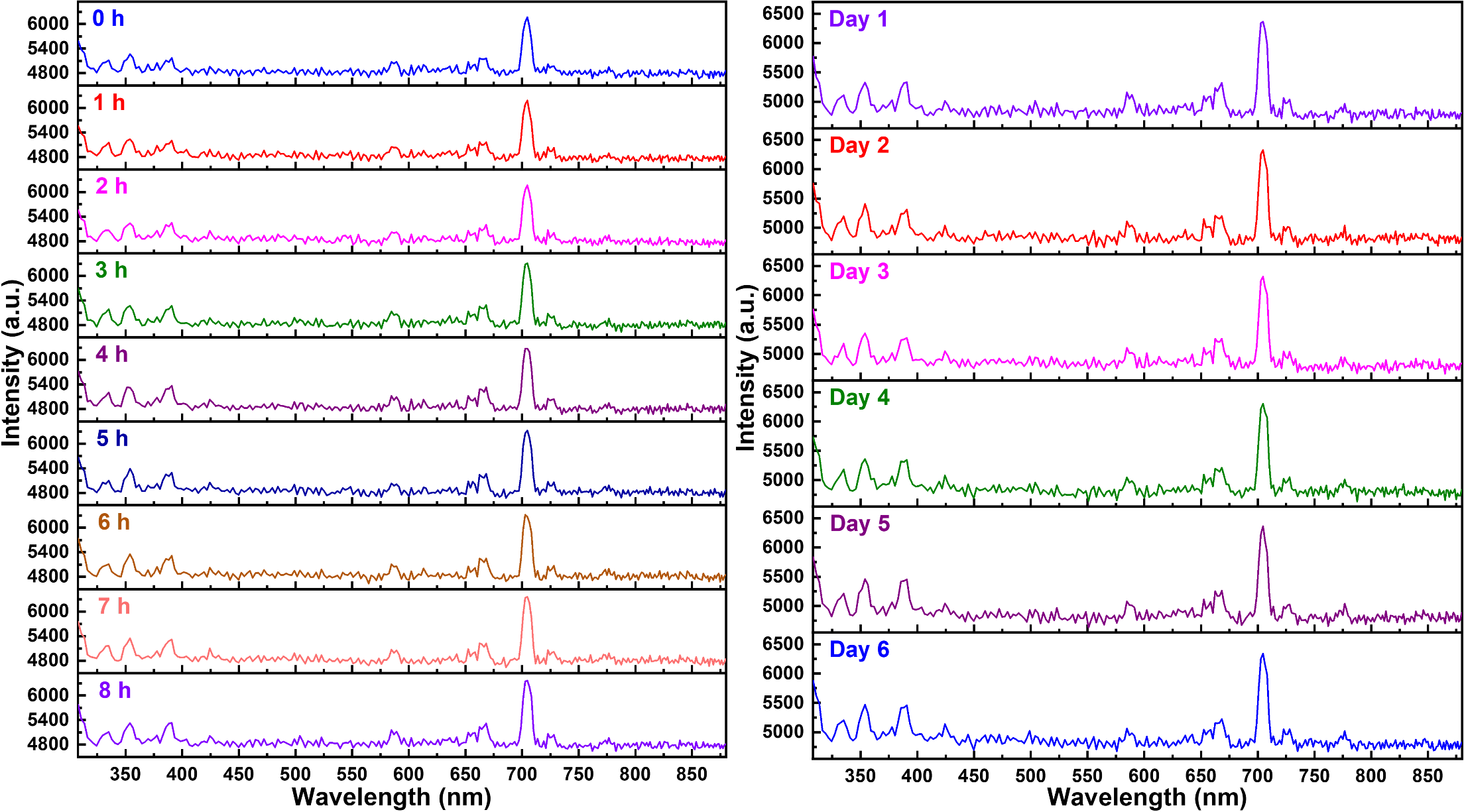


**Figure S2.** The hourly OES of the plasma jet generated by Device 4 during 8 hours of continuous operation on Day 1 and the OES on Days 1-6 after at least 20 minutes of continuous operation each day.


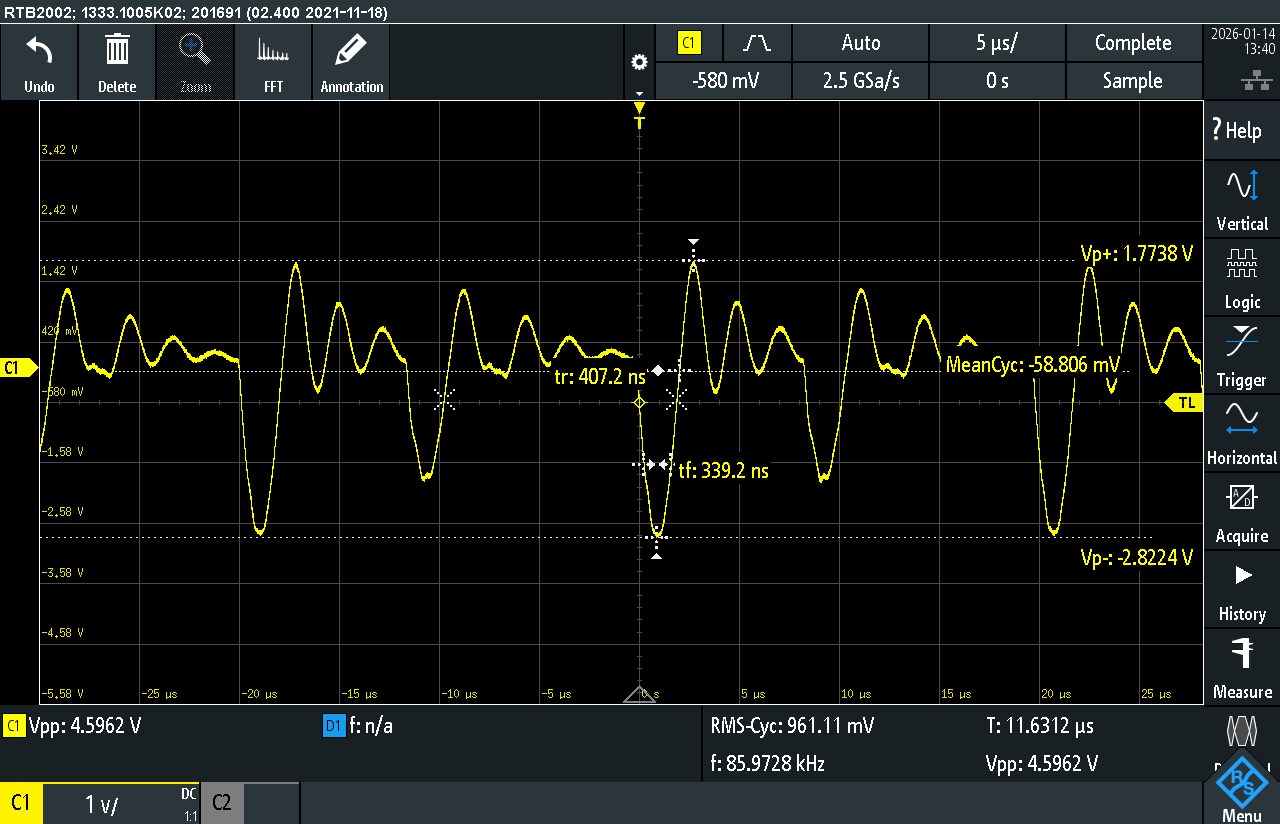


**Figure S3.** Output voltage waveform of the custom power supply using a high voltage 500:1 probe (Micsig DP10013 Differential Probe). The maximum output voltage was limited by the operating voltage range of the probe. Therefore, we only tested the power supply output when a stable and visible plasma ignition is observed at ~480V_RMS_. The output voltage waveform is consistent with the one specified in the datasheet of ZS1052(H). The output voltage of ~900 V_RMS_ used for disinfection experiments is independently measured using a digital multimeter.
